# Phosphorus adsorption characteristics and release risk in saline soils: a case study of Songnen Plain, China

**DOI:** 10.3389/fpls.2023.1302763

**Published:** 2023-12-06

**Authors:** Yan Jiang, Qiuliang Yan, Tonglinxi Liu, Yifan Xu, Xing Han, Xiulan Ma, Yujun Wang

**Affiliations:** ^1^ College of Resources and Environment, Jilin Agricultural University, Changchun, China; ^2^ Institute of Animal Nutrition and Feed Sciences, Jilin Academy of Agricultural Sciences, Gongzhuling, China; ^3^ Jilin Huan Zhi Technology Co., LTD., Changchun, China

**Keywords:** phosphorus, salt -alkaline soil, environmental factors, adsorption capacity, release risk

## Abstract

**Introduction:**

The Songnen Plain is one of the three major saline-alkali areas in China, covering a vast area, where drought and overgrazing have exacerbated the salinization trend, and will have great potential for development if utilized rationally. Phosphorus, as one of important soil nutrients, plays a crucial role in plant growth. How to minimize its loss and migration has become a current research hotspot. The objective of the present study was to elucidate the adsorption properties of phosphorus in soils affected by salinization and to establish the correlation between the potential for phosphorus release and soil properties.

**Methods:**

A batch treatment test was conducted in this study using three soils with the various salinization degrees to examine the impact of environmental factors on the adsorption properties and potential release of phosphorus.

**Results and discussion:**

It was found that the maximum phosphorus adsorption by the three salinization soils in 0-360 minutes accounted for 86.8%-90.5% of the total adsorption capacity; the equilibrium adsorption capacity was: HS> MS> LS. In cases where the phosphorus level in the surrounding liquid is low, the three levels of salinized soils exhibited varying levels of phosphorus discharge, with the adsorbent acting as the origin of contaminants. The Pseudo-second-order model kinetics and Langmuir equation can well describe the adsorption process, and the adsorption process is spontaneous heat absorption with entropy increase. Increasing the pH led to an increase in the adsorption of phosphorus from the three salinized soils. Additionally, the adsorption was enhanced by introducing varying concentrations of Na^+^, Ca^2+^, and Al^3+^ to the background solution. The phosphorus eutrophication release risk (ERI) demonstrated a gradual decline as temperature increased. Correlation analysis revealed a noteworthy positive correlation between TN, TP, and ERI, as well as a significant negative correlation between CEC, K^+^, and ERI. Furthermore, there was a highly significant negative correlation between coarse silt and fine silt. Considering local climatic and environmental factors is crucial for controlling the adsorption capacity of phosphorus in various salinized soils, as it can unveil the mechanism of phosphorus adsorption and impact its migration and release risk.

## 1 Introduction

Phosphorus, a vital component for the development of plants and human endeavours, serves a crucial function in upholding the equilibrium of ecosystems and the cycling of nutrients (Amarh et al., 2021; Amadou et al., 2022). Saline soil is a widely distributed agrotype on earth. Furthermore, the saline soil of Songnen Plain in western Jilin Province is one of the three regions in the world with concentrated different degrees of salinization and relatively low basal fertility. The Songnen Plain has reached an area of 4.97×10^6^ ha, and the surface is flat and suitable for farming (Ren et al., 2022). Therefore, clarifying the risk of phosphorus release in saline soils is of great significance for agricultural development as well as for promoting food security. In saline soils, phosphorus exists in both inorganic and organic forms, with the majority being inorganic phosphorus, which makes up 60%-80% of the overall phosphorus content in the soil (Zhao et al., 2023). Soil fertility can be assessed by considering the level of phosphorus content, which is an essential indicator. Local farmers have a habit of applying an excessive amount of phosphorus fertilizers all at once (Zhang et al., 2022). Global utilization of phosphate fertilizers is estimated to be only 10%-20% based on statistical data, the excessive accumulation of phosphorus fertilizer poses a risk of phosphorus loss (Zhao et al., 2021).The presence of phosphorus transport and release, a significant contributor to surface pollution, presents a substantial risk to the current deterioration of the environment (Hahn et al., 2012; Tu et al., 2019).

Adsorption is the main way of soil phosphorus fixation, and phosphorus fertilizer adsorb into the soil is in a dynamic balance between plant uptake and soil adsorption and desorption. Soil adsorption and desorption exhibit contrasting behaviors, with desorption referring to the detachment of elements from the soil surface to the solid state. Adsorption and desorption behavior primarily regulate the potential for phosphorus transportation and release within the soil. It has been shown that soil pH, metal elements aluminium and calcium can significantly affect phosphorus adsorption in soil (Jing et al., 2024). The addition of Fe(II) biochar to saline soil enhanced its phosphate adsorption capacity (Wu et al., 2020), and addition of biochar to black soil improved the storage capacity of organic phosphorus (Zhao et al., 2022; Ilori et al., 2023). In contrast, the mode of adsorption of phosphorus by black soil is mainly monolayer chemisorption, which is related to electrostatic repulsion and oxygen-containing functional groups (Xue et al., 2023). Non-electrostatic adsorption (complexation, ion exchange) is the main way in which metal ions affect phosphorus adsorption by soils. (Bao et al., 2023). The presence of anions in the soil significantly affects the adsorption capacity, as anions compete with PO_4_^3-^ for binding sites (Jin et al., 2006; Yang et al., 2019).

The pollution caused by phosphorus is related to the TP content of the soil (the sum of organic and inorganic phosphorus) and the specific form of phosphorus in the soil. The Hedley method for classifying phosphorus is a valid approach considering both organic and inorganic phosphorus. This method has gained international recognition from foreign researchers. Dinkler et al. (2021) primarily utilize the PSI and DPS methods to evaluate the potential for phosphorus transport and release. PSI is commonly employed to assess the quantity of phosphorus in the soil that may be discharged into the liquid phase (Chen et al., 2003), while DPS indicates the extent to which phosphorus has been adsorbed by the soil, serving as an indicator of its capacity to absorb and retain phosphorus (Huang et al., 2004). Nevertheless, the potential for transportation and discharge of phosphorus from soils also significantly differs depending on various environmental factors (Qin et al., 2023). Several studies have indicated that temperature, acidity level, type of ions, and concentration of ions can all exert an influence on this phenomenon (Zhu et al., 2015; Sharma et al., 2023). Therefore, it is important to investigate the effects of different salinized soils and their environmental factors on phosphorus adsorption capacity to assess and predict the risk of phosphorus transport release. (Chen et al., 2015).

In this study, soil samples were collected in Daan City, Jilin Province using contour sampling method. Three different salinized soils were selected for salinity level-based analysis. The objectives of this study are (1) to clarify the effects of different salinized soils on phosphorus adsorption. (2) To demonstrate the effect of different environmental factors on phosphorus adsorption. (3) To assess the risk of phosphorus transport and release in soil. To provide accurate reference for effective control of phosphorus pollution risk in different salinized soils for future sustainable development.

## 2 Materials and methods

### 2.1 Sample collection and processing

#### 2.1.1 Study area

**Figure 1:**
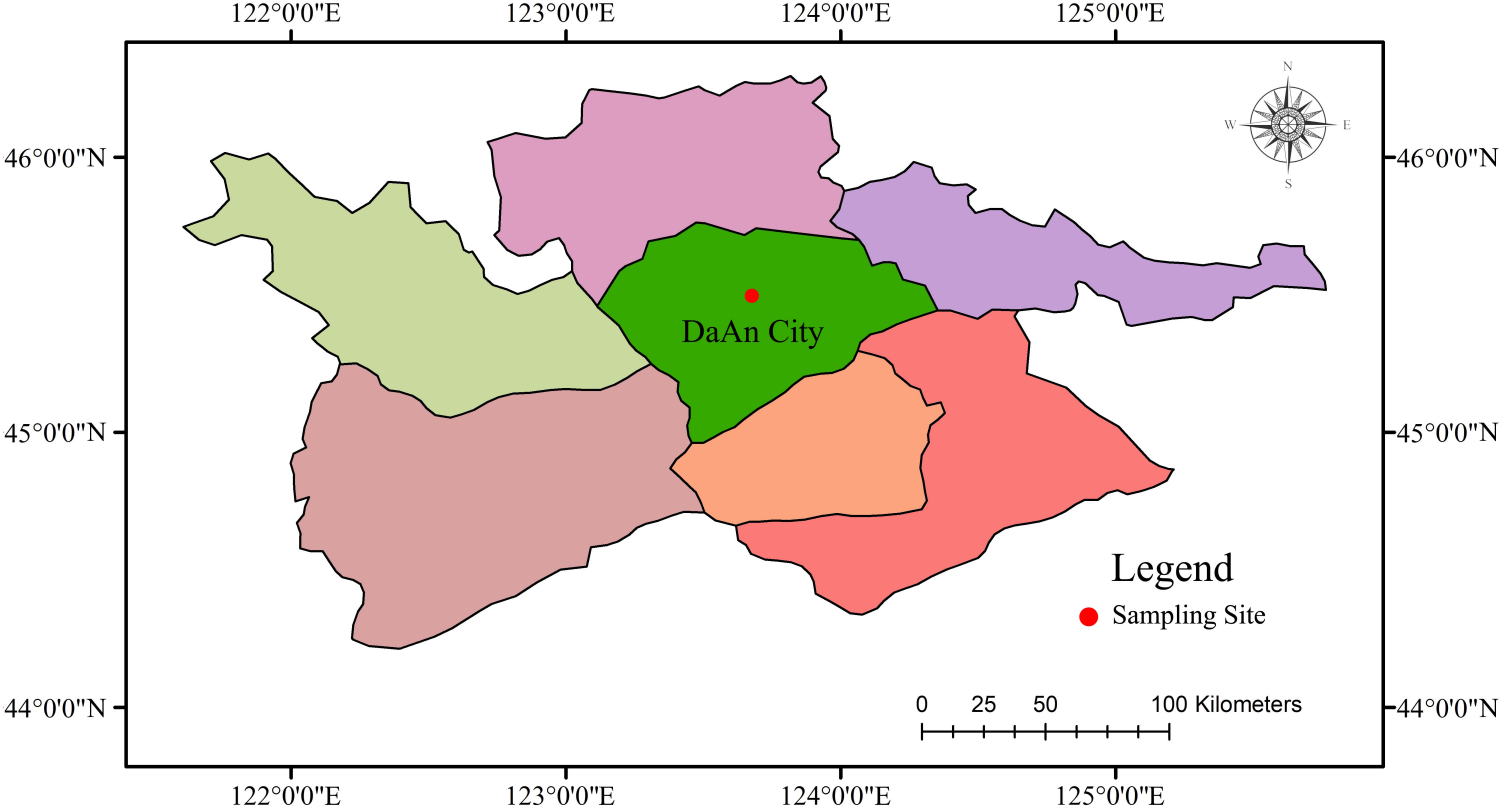
Distribution map of study area.

The soil examined in this research was collected from Xishieripu, Lesheng Township, Anguang Town, Daan City, Jilin Province, China (East Longitude 123°18′-125°32′, North Latitude 44°22′-45°36′). The study area has a mesothermal monsoon climate with hot summers and cold, dry winters. The average annual temperature is 4.3°C, and the average annual rainfall is 400 mm. Due to the variation in altitude, the salts on the soil surface are distributed unevenly, leading to varying degrees of soil salinization (Wang et al., 2009; Imseng et al., 2019). Approximately 59% of the entire soil area in Daan City belongs to saline-alkali soil. Of them, around 74% is severely affected by salinization, significantly impacting both the local ecosystem and agricultural production activities. **Figure 1** displays the map of the study area in great detail. The information in the **Figure 1** was from the Ministry of Natural Resources of the People’s Republic of China (https://www.mnr.gov.cn/).

#### 2.1.2 Sample processing

Collect the top 0-20 cm layer of soil, eliminate any contaminants present, and allow it to air dry in a well-ventilated area. Subsequently, pulverize the soil using a 100-mesh sieve and store it in a self-sealing bag for future use. Three saline soils with different degrees of salinization were selected as test soils by determining the pH(FE20-FiveEasy, Switzerland), conductivity(DDSJ-319L, China) and salt content(JC-TEC, China) of the soils. The fundamental physicochemical characteristics of the examined soil were assessed using the approach outlined in the publication ‘Routine Analytical Methods of Soil Agrochemistry’ (Bao et al., 1988), and the findings were presented in **Table 1** and **Table 2**.

**Table 1:** Soil physicochemical properties measurement.

Soil type	pH	EC (mS/cm)	Saltiness (g/kg)	SOM (g/kg)	CEC (cmol/kg)	TN (g/kg)	TP (g/kg)	Mechanical composition(%)			
								sand	coarse silt	fine silt	clay
LS	(8.59 ± 0.24)a	(17.60 ± 0.69)a	(1.90 ± 0.02)a	(13.04 ± 0.42)b	(14.10 ± 0.41)a	(0.38 ± 0.02)c	(0.85 ± 0.03)c	(28.80 ± 0.41)a	(15.77 ± 0.14)a	(6.33 ± 0.15)a	(49.10 ± 0.33)c
MS	(9.33 ± 0.11)b	(19.40 ± 0.61)b	(3.09 ± 0.05)b	(12.38 ± 0.33)b	(18.40 ± 0.31)b	(0.25 ± 0.01)b	(0.73 ± 0.02)b	(29.40 ± 0.25)a	(23.26 ± 0.19)b	(9.54 ± 0.18)b	(37.80 ± 0.14)b
HS	(10.97 ± 0.33)c	(25.00 ± 0.66)c	(5.12 ± 0.11)c	(10.89 ± 0.36)a	(21.30 ± 0.35)c	(0.17 ± 0.01)a	(0.66 ± 0.03)a	(34.50 ± 0.49)b	(28.51 ± 0.22)c	(11.79 ± 0.21)c	(25.20 ± 0.23)a

The data in the table are mean ± standard deviation; within columns, means followed by the same letter are not significantly different(p<0.05).

**Table 2:** Content of water-soluble salt-based ions in saline soils.

Soil type	Na^+^	K^+^	Ca^2+^	Mg^2+^	CO_3_^2-^	HCO_3_^-^	Cl^-^	SO_4_^2-^
	(g/kg)							
LS	(5.68 ± 0.15)a	(1.21 ± 0.08)a	(0.09 ± 0.01)a	(0.14 ± 0.01)a	(3.61 ± 0.09)a	(0.31 ± 0.01)a	(0.09 ± 0.01)a	(0.26 ± 0.02)a
MS	(7.03 ± 0.15)b	(2.18 ± 0.05)b	(0.20 ± 0.01)b	(0.22 ± 0.01)b	(8.72 ± 0.13)b	(1.19 ± 0.11)b	(0.71 ± 0.04)b	(0.32 ± 0.01)b
HS	(15.52 ± 0.14)c	(3.09 ± 0.12)c	(0.35 ± 0.02)c	(0.49 ± 0.01)c	(8.84 ± 0.16)b	(1.66 ± 0.06)c	(1.02 ± 0.03)c	(0.38 ± 0.01)c

The data in the table are mean ± standard deviation; within columns, means followed by the same letter are not significantly different(p<0.05).

### 2.2 Soil phosphorus fractionation

To determine Ca_2_-P, Al-P, O-P, Fe-P, O-Al-P, O-Fe-P, and Ca_10_-P, the research employed the Hedley (Hou et al., 2018; Yan et al., 2018) grading technique for assessing phosphorus levels in soils (The methodology is described in the **Supplementary Material**).

### 2.3 Phosphorus adsorption experiment

#### 2.3.1 Adsorption kinetics experiment

50mL polyethylene centrifuge tubes were used to hold 1.0000 ± 0.0005g of the tested soil. Then, 20mL of phosphorus solution with a concentration of 100mg/L was added. A background solution of NaCl with a concentration of 0.01mol/L was also included. Oscillate the tubes away from light at a constant temperature of 298K. Samples were taken at 0, 30, 60, 120, 240, 360, 480, 720, and 1440 minutes. To inhibit microbial activity, three drops of chloroform were added to each treatment. Soil solutions are centrifuged for 10 min at 10000 rpm and the supernatant was filtered (0.45 μm). This filtration enabled colorimetric analysis using the ascorbic acid-molybdenum phosphoramidite blue method.

#### 2.3.2 Adsorption isotherm experiments

To conduct the test described in section 2.3.1, modify the concentration of the phosphorus solution to 10, 20, 40, 60, 80, 100, and 150 mg/L. Maintain a constant temperature of 288, 298 and 308 K, ensure protection from light, and subsequently analyze the phosphorus content in the supernatant once adsorption equilibrium is achieved.

#### 2.3.3 The effect of environmental factors on phosphorus adsorption

The test method 2.3.2 involved adjusting the pH of the phosphorous solution was adjusted to 3,5,7,and 9 with 0.1M HCl or 0,1 NaOH. As for the background solution, three cations (Na^+^, Ca^2+^, Al^3+^) were chosen with concentrations of 0.01, 0.05, 0.1, 0.15, 0.2 mol/L, respectively. This was done to examine the impact of ionic strength and different ions on the adsorption capacity of phosphorus.

The above experiments were set up with 3 groups of replications.

### 2.4 Data analysis methods

PSI:


(Eq.1) $$\text{PSI}=\frac{\text{X}}{lgC}$$

The quantity of absorbed phosphorus was denoted as X (mg/100g), and the phosphorus adsorption index can be computed as PSI = X/lg C. In this equation, C signifies the concentration of dissolved phosphorus in the filtrate (umol/L), and the PSI is measured in units of (mg P/100g)/(umol/L).

DPS:


(Eq.2) $$DPS=\frac{100{\text{P}}_{\text{ox}}}{\left({\text{Al}}_{\text{ox}}+{\text{Fe}}_{\text{ox}}\right)/2}\times 100\%$$

Pox-active phosphorus, mmol/kg; Alox-active aluminum, mmol/kg; Feox-active iron, mmol/kg.

ERI:

Huang (Huang et al., 2004) suggested using the DPS to PSI ratio as a means of determining the soil (ERI).


(Eq.3) $$\text{ERI}=\frac{\text{DPS}}{\text{PSI}}\times 100\%$$

The statistical analysis of the data was performed using IBM SPSS Statistics 23, where a significance level of p< 0.05 was considered. Additionally, the adsorption and desorption of phosphorus in various salinized soils were fitted using Origin 8.5 software (Origin Lab, Northampton, MA, USA).

## 3 Results and discussion

### 3.1 Morphological analysis of phosphorus fractionation

Due to the characteristics of saline soils and environmental features, there is a large influence on phosphorus effectiveness in soils. Soil with high activity phosphorus is characterized by effective state P (Ca_2_^-^P), while soil with medium activity phosphorus is characterized by retarded state P (Al-P and Fe-P), and soil with low activity phosphorus is characterized by closed storage state P (O-P and Ca_10_-P) (Dari et al., 2016). The results of phosphorus grading of different saline soils were showed in **Table 3**. The TP content of the three saline soils was determined as follows: HS had the lowest content, MS, and LS had the highest content. The range of effective state P (Ca_2_-P) content was between 22.11 and 35.72 mg/kg. The Al-P content in severely saline soil reached 151.71 mg/kg, which accounted for 23.0% of TP, indicating the highest closed storage state. As soil salinization deepens, the concentration of Ca^2+^ rises, leading to an increase in phosphorus adsorption in salinized soil (Tang et al., 2014). However, some of the available phosphorus is transformed into closed storage phosphorus, which cannot undergo adsorption desorption in the soil (Tian et al., 2020). Mildly salinized soil exhibited the highest level of available phosphorus, making it a promising phosphorus source for plants (Zhan et al., 2019). This also implies that the transportation of phosphorus intensifies and its potential for release becomes greater.

**Table 3:** Phosphorus fractionation in three soil differemt salinity levels.

Phosphorus form	Ca2-P	Al-P	O-P	Fe-P	O-Al-P	O-Fe-P	Ca10-P	TP
LS/(mg/kg)	(35.72 ± 1.28)c	(20.25 ± 0.08)c	(72.63 ± 2.57)c	(252.29 ± 6.79)b	(365.71 ± 7.45)c	(0.19 ± 0.08)a	(103.57 ± 0.85)b	(850.36 ± 16.75)c
MS/(mg/kg)	(28.77 ± 3.45)b	(16.72 ± 0.02)b	(62.35 ± 5.52)b	(246.78 ± 4.14)b	(272.33 ± 2.08)b	(0.47 ± 0.06)b	(102.86 ± 0.49)b	(730.28 ± 20.32)b
HS/(mg/kg)	(22.11 ± 1.17)a	(14.69 ± 0.12)a	(49.57 ± 3.24)a	(216.59 ± 3.98)a	(258.26 ± 1.96)a	(0.13 ± 0.02)a	(99.04 ± 0.66)a	(660.39 ± 12.33)a

The data in the table are mean ± standard deviation; within columns, means followed by the same letter are not significantly different(p<0.05).

### 3.2 Adsorption characteristics of phosphorus

#### 3.2.1 Adsorption kinetics

**Figure 2:**
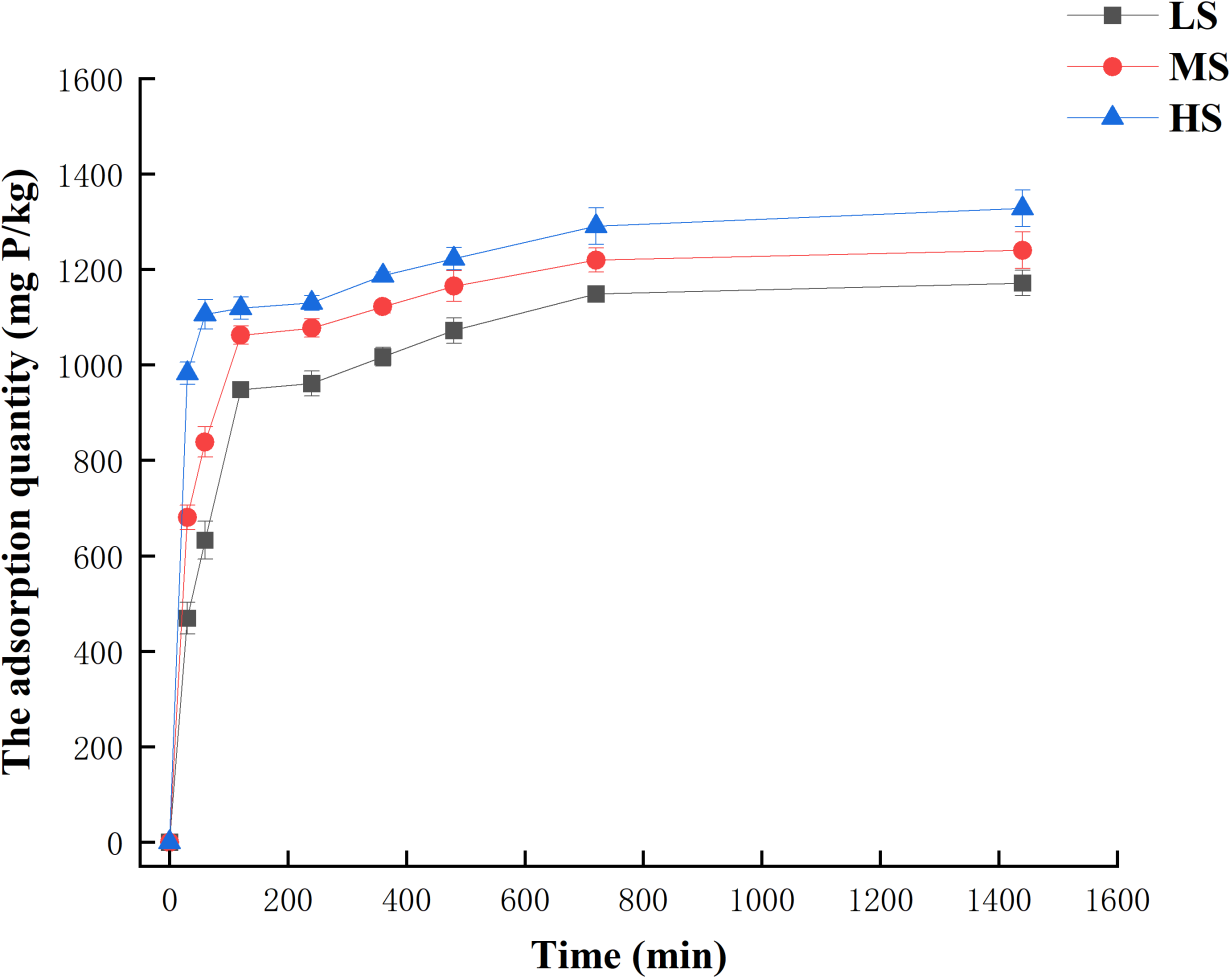
Adsorption kinetic of phosphorus.

The adsorption of phosphorus in various salinized soils changed over time when the initial phosphorus concentration was 100 mg/L. As we can see from **Figure 2**. The process of phosphorus adsorption by the soil involved multiple stages, with the initial 0-360 minutes being a rapid adsorption phase. During this phase, the soil’s phosphorus adsorption capacity increased significantly over time, accounting for 86.8%, 90.5%, and 89.4% of the maximum adsorption. Subsequently, from 360 to 480 minutes, the adsorption stage transitioned into a slower phase, with a smaller increase in adsorption amount. In order to ensure that the adsorption reached the full equilibrium, the equilibrium adsorption amount of phosphorus from the three kinds of test soils was set at 720 min as the equilibrium time for adsorption. The adsorption equilibrium of phosphorus by the three tested soils followed the order: LS< MS< HS.

The present study utilized the Pseudo-first-order model (**Table S1**) and Pseudo-second-order (**Table S1**) model to fit the kinetic characteristics of phosphorus adsorption in various salinized soils. **Table 4** displays the fitting parameters of the various equations. Upon examining the correlation coefficients r in the table, it becomes apparent that the two equations are more adept at accommodating the adsorption kinetic data of phosphorus, and all of them attain a remarkably significant level (p< 0.01). **Table 4** reveals that the Pseudo-second-order model provided a better fit for the equilibrium adsorption of light, medium, and HS. The correlation coefficient (r) exceeded 0.993, indicating a close approximation to the actual value. Moreover, this equation encompasses various adsorption processes, including surface adsorption, diffusion through the external liquid film, and particle diffusion.

**Table 4:** Fitting parameters for adsorption kinetics in different salinized soils.

Soil type	Pseudo-first-order model			Pseudo-second-order model		
	*q_e1_*	*k_1_*	*r*	*q_e2_*	*k_2_*	*r*
LS	927.4	173.4	0.765**	1193.8	1.8	0.993**
MS	1050.6	196.3	0.870**	1236.9	3.1	0.997**
HS	1170.6	218.4	0.962**	1254.5	8.7	0.991**

** Indicate statistical significance at p ≤ 0.01.

#### 3.2.2 Adsorption isotherm

**Figure 3:**
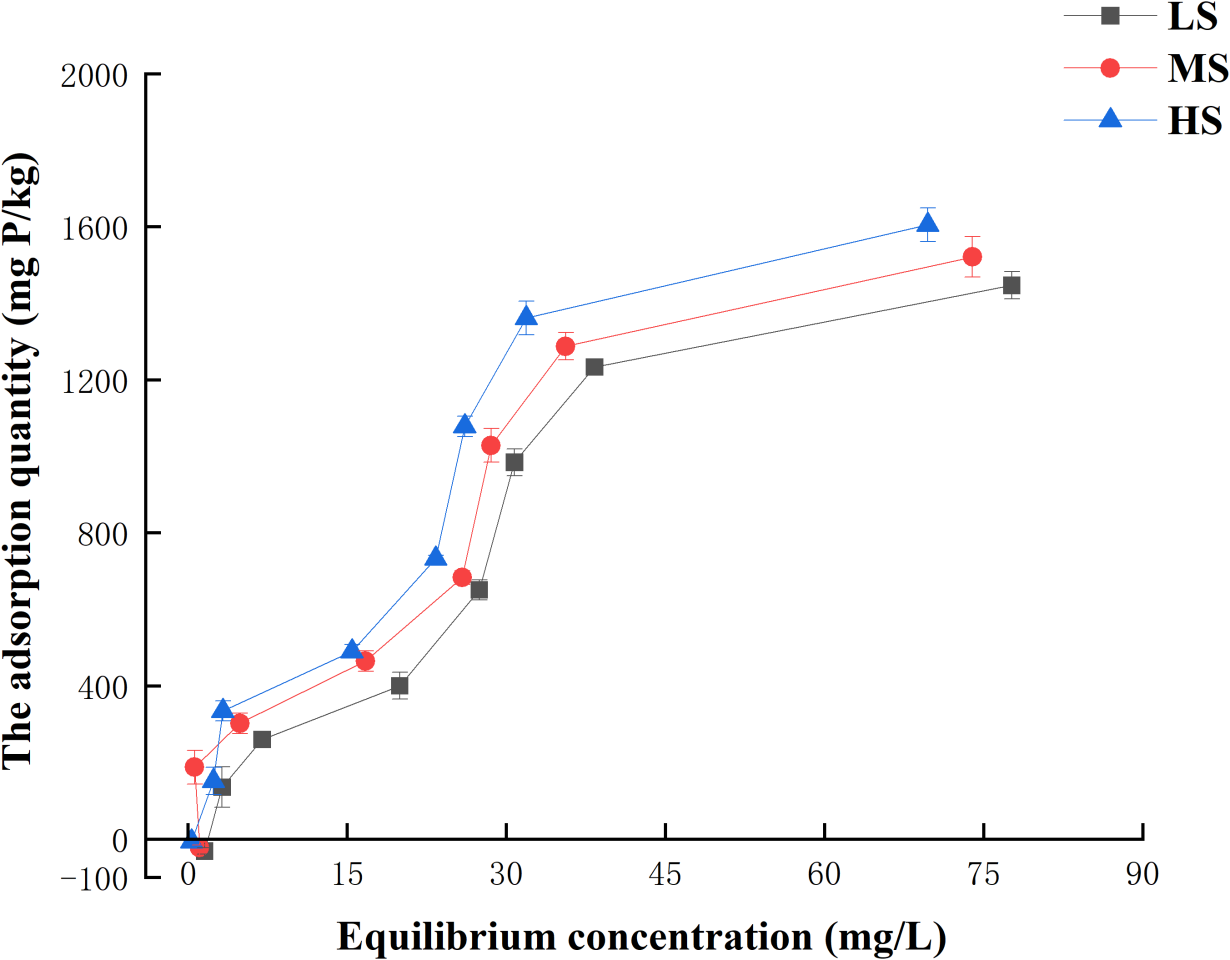
Adsorption isotherms of phosphorus.

Plotting adsorption isotherms based on the amount of phosphorus adsorbed by different salinized soils. As shown in **Figure 3**. At a background liquid concentration of 0 mg/L, the adsorption quantities in three salinized soils were -31.5, -22.0, and -6.9 mg/kg, respectively. During this period, the soil acted as the ‘origin’ of phosphorus, exhibiting varying levels of phosphorus release among the three types of salinized soils. With the increase in the initial phosphorus concentration, there was a gradual rise in the amount of adsorption, ultimately transforming the soil into a phosphorus ‘sink’. Increasing the phosphorus background concentration to 150 mg/L resulted in equilibrium adsorption amounts of 1331.8 mg/kg for Mild salinization, 1389.1 mg/kg for Moderate salinization, and 1462.8 mg/kg for HS. We compared the available studies (**Table S2**). Usually, we categorize the process of phosphorus adsorption in soil into the physical adsorption process and chemical adsorption process. At low phosphorus concentrations, chemical adsorption dominated and adsorption was completed rapidly. At high phosphorus concentrations, the adsorption sites became saturated, the chemical adsorption slowed down rapidly, and physical adsorption dominated.

In this study, the Langmuir equation (**Table S1**) and the Freundlich equation (**Table S1**) were used to perform isothermal fitting of three different salinized soils for phosphorus adsorption. The Langmuir equation is a theoretically derived equation that assumes the presence of numerous adsorption activity centers on the solid surface. When all the adsorption activity centers are occupied, it represents the ideal scenario of an adsorption monomolecular layer with no interaction among them. In contrast, the Freundlich equation, an empirical formula, does not account for maximum adsorption capacity. The Freundlich equation is applicable to physical adsorption, chemical adsorption, and solution adsorption, and is commonly utilized to describe multilayer adsorption. **Table 5** displayed the fitted parameters. The correlation coefficients obtained from fitting the Langmuir and Freundlich equations show a highly significant difference. However, the correlation coefficient ‘r’ for the Langmuir equation is slightly higher than that of the Freundlich equation. The adsorption of phosphorus by saline soils can be explained by monomolecular layer adsorption (Langmuir, 1918). The adsorption sites on the soil surface are uniformly distributed, and the dominant type of adsorption is chemisorption. The Langmuir equation provides a better representation of the adsorption behavior of phosphorus in salinized soil.

**Table 5:** Adsorption isotherm parameters of different salinized soils.

Soil type	Langmuir model			Freundlich model		
	*k_L_*	*q_m_*	*r*	*k_F_*	*1/n*	*r*
LS	199.8	2807.4	0.929**	78629.1	0.8268	0.917**
MS	327.6	3264.8	0.957**	131137.4	0.7482	0.944**
HS	422.1	3971.9	0.963**	190407.1	0.6658	0.948**

** Indicate statistical significance at p ≤ 0.01.

According to Li et al. (2023), a positive value of k_L_ in the Langmuir equation suggests the spontaneous progression of the reaction. Furthermore, a higher k_L_ signifies a greater extent of spontaneous reaction. The analysis of phosphorus adsorption in the three soils led to the conclusion that k_L_ showed an increase corresponding to the level of soil salinization, suggesting a direct relationship between the degree of salinization and the strength of phosphorus adsorption in the soils. Additionally, q_m_ exhibited a similar increase as the salinization degree rose. The increase in clay content of the three tested soils may be attributed to the deepening of soil salinization, leading to an augmentation in specific surface area and consequently exchange capacity. As a result, the adsorption capacity of phosphorus becomes stronger. The LS had a lower saturated adsorption capacity compared to the MS, which in turn had a lower capacity than the HS. The difference in solid-liquid partitioning properties and binding forces between the phosphorus already bound to the solid-phase medium and phosphorus adsorbed in the three salinized soils leads to phosphorus release at low background fluid concentrations (Jin et al., 2013).

### 3.3 Phosphorus adsorption on different environmental factors

#### 3.3.1 Effect of temperatures on the adsorption of phosphorus

At various temperature conditions (288K, 298K, 308K), **Table 6** exhibits the absorption of phosphorus by the three providing soils. We can see that the saturated adsorption of phosphorus (q_m_) of the three test soils gradually increased with higher temperature. Compared to 288 K, the saturated adsorption of phosphorus was enhanced by 15.8%, 14.8%, and 13.6% for mild, moderate, and HS, respectively, at 308K. Additionally, the three test soils exhibited an increase in the saturated adsorption of phosphorus at varying temperatures. The correlation coefficients of the adsorption isotherms fitted using Langmuir’s equation all reached the highly significant correlation level.

**Table 6:** Langmuir parameters at different temperature conditions.

Soil type	Temperature (*T/K*)	*k_L_*	*q_m_*	*r*
LS	288	159.8	2714.1	0.921**
	298	199.8	2807.4	0.929**
	308	369.9	2821.6	0.956**
MS	288	257.2	2841.3	0.944**
	298	327.6	3264.8	0.957**
	308	507.4	3281.5	0.955**
HS	288	338.3	3160.3	0.957**
	298	422.1	3971.9	0.963**
	308	620.2	4203.4	0.958**

** Indicate statistical significance at p ≤ 0.01.

The arrangement of organic and inorganic elements within the three salinized soils is quite intricate, and variations in temperature significantly influence the biochemical processes occurring within the soils (Yan et al., 2017; Yuan et al., 2020). **Table 7** displayed the thermodynamic parameters that were calculated for the three test soils at various temperatures. We can see that the Gibbs free energy of phosphorus adsorption ΔG<0 (**Table S1**) for the three test soils of mild salinity, moderate salinity, and severe salinity indicates that phosphorus adsorption is spontaneous under the standardized state (Yan et al., 2015), and the ΔG increases with temperature, indicating that the high temperature facilitates adsorption proceed; ΔH>0 (**Table S1**) indicates that the adsorption reaction was a heat-absorbing process. High temperature favors adsorption. ΔS>0 (**Table S1**) indicates entropy increase, exothermic entropy increasing spontaneous reaction process, which were consistent with the adsorption thermodynamic results (Hu et al., 2017). Phosphorus immobilization in the soil is favored by high temperature conditions, while lower temperatures weaken phosphorus adsorption capacity and enhance phosphorus transport activity.

**Table 7:** Thermodynamic parameters of phosphorus adsorption under different temperature conditions.

Soil type	*T/*K	Δ*G/* kJ/mol	Δ*H/* kJ/mol	Δ*S/* kJ^/^mol^/^k
LS	288	-12.150	31.11	0.15
	298	-13.125		
	308	-15.142		
MS	288	-13.289	25.14	0.13
	298	-14.349		
	308	-15.951		
HS	288	-13.945	22.43	0.13
	298	-14.977		
	308	-16.466		

#### 3.3.2 Effect of pH on the adsorption of phosphorus in soil

**Figure 4:**
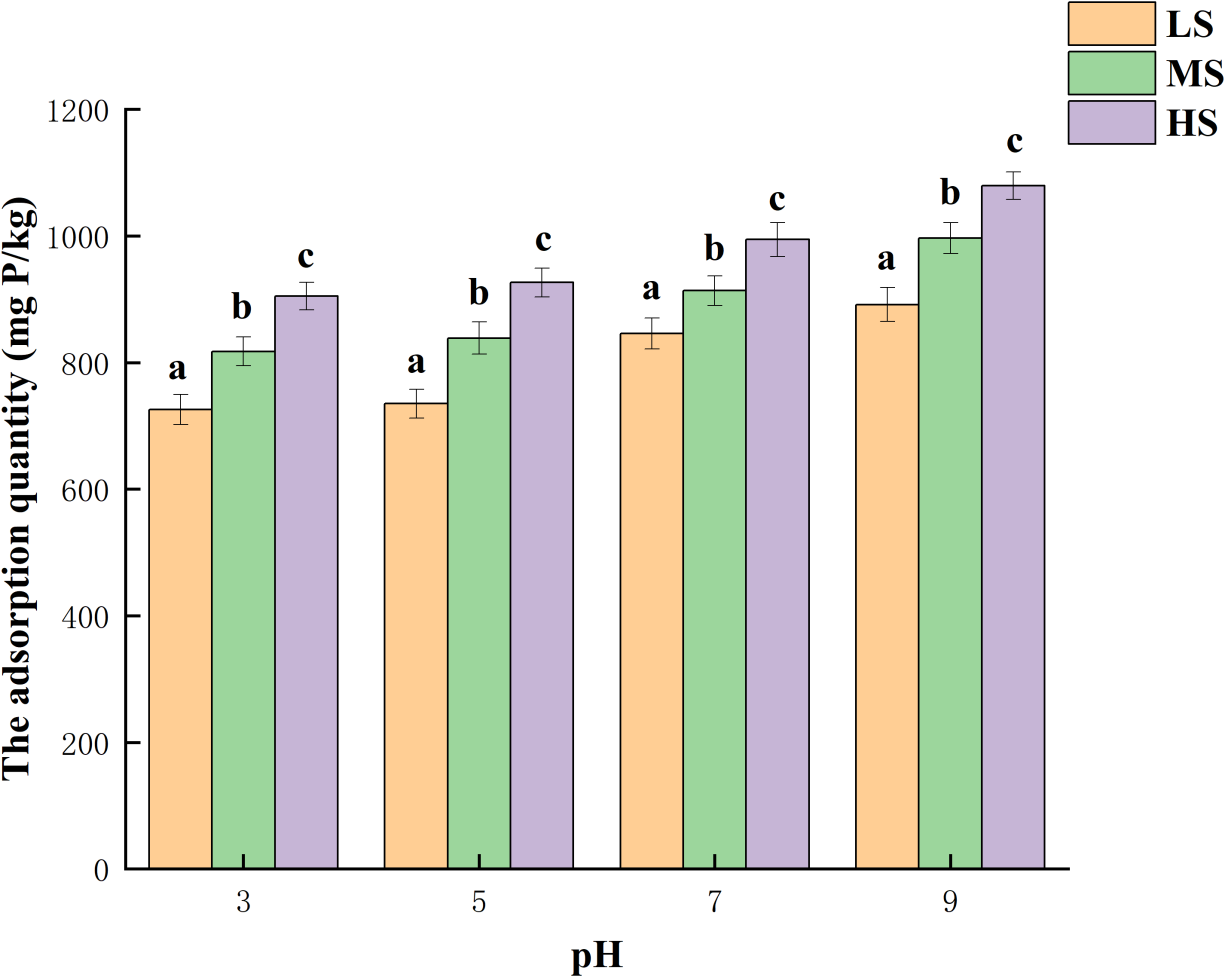
The impact of varying pH levels on the absorption of phosphorus. Different lowercase letters represent significant differences.

**Figure 4** illustrates the quantity of phosphorus adsorption in the three salinized soils when the pH is different. In the test, it is observed that the phosphorus concentration was 100 mg/L, and the adsorption of phosphorus increases as the pH level increases within the tested range. Phosphorus adsorption exhibited a smaller variation at pH 3-5, whereas a more significant variation in adsorption occurred with an increase in pH to 5-9. Compared to pH 3, the adsorption quantity experienced a 22.9%, 21.9%, and 19.3% increase at pH 9. The possible explanation could be that a decrease in pH leads to a higher occupancy of the adsorption sites in the experimental soil by H^+^ ions (Chen et al., 2015), resulting in reduced phosphorus adsorption. Conversely, as the solution pH gradually increases, the competition between H^+^ ions and PO_4_^3-^ ions for the active sites on the surface of the saline soil diminishes, thereby enhancing the adsorption capacity of phosphorus in the experimental soil (Amarh et al., 2021). The influence of background solution pH on phosphorus sequestration in soil is evident (Huang et al., 2016). The rise in soil pH led to an increase in the presence of PO_4_^3-^ in the soil, consequently enhancing the soil’s capacity for sequestering phosphorus (Yang et al., 2019).

#### 3.3.3 Effect of Na^+^, Ca^2+^, and Al^3+^ concentrations on phosphorus adsorption

**Figure 5:**
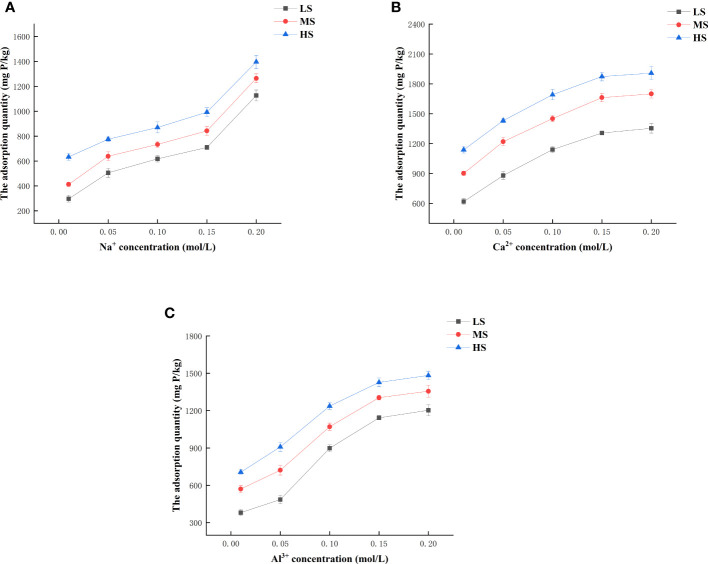
The impact of different concentrations of Na^+^ **(A)**, Ca^2+^ **(B)**, and Al^3+^ **(C)** on the adsorption of phosphorus.

The composition and ratio of Na^+^ and Ca^2+^ in the soil are associated with the level of soil salinity, and can serve as an indicator for assessing the soil. Al^3+^ in soil can increase soil nutrients and improve soluble salt composition to different degrees, and Al_2_(SO_4_)_3_ is usually used for saline soil improvement. **Figure 5** shows the effects of Na^+^, Ca^2+^, and Al^3+^ concentrations on phosphorus adsorption, respectively. The rise in various ion concentrations resulted in a similar pattern of phosphorus adsorption by the three types of soils provided, with a gradual increase. Additionally, as the ion valence increased under the same ion concentration, the adsorption capacity of the three types of saline soils exhibited an initial increase followed by a subsequent decrease. Possible explanations include the Na^+^ reducing the electrostatic repulsion between the adsorbed phosphorus, resulting in higher phosphorus adsorption. Additionally, the elevated Ca^2+^ levels may react with phosphorus in the solution, forming calcium hydroxyphosphate in alkaline conditions, thereby enhancing phosphorus adsorption in saline soils. Furthermore, the ligand-exchange reaction between Al^3+^ and PO_4_^3-^ can also facilitate phosphorus adsorption. Na_3_PO_4_, Ca_10_(PO_4_)_6_(OH)_2_, and AlPO_4_ precipitates can be formed with OH^-^ in solution at the same ionic concentration of Na^+^, Ca^2+^, and Al^3+^, respectively. This process enhances the adsorption of phosphorus by saline soil.

#### 3.3.4 Adsorption mechanism

**Figure 6:**
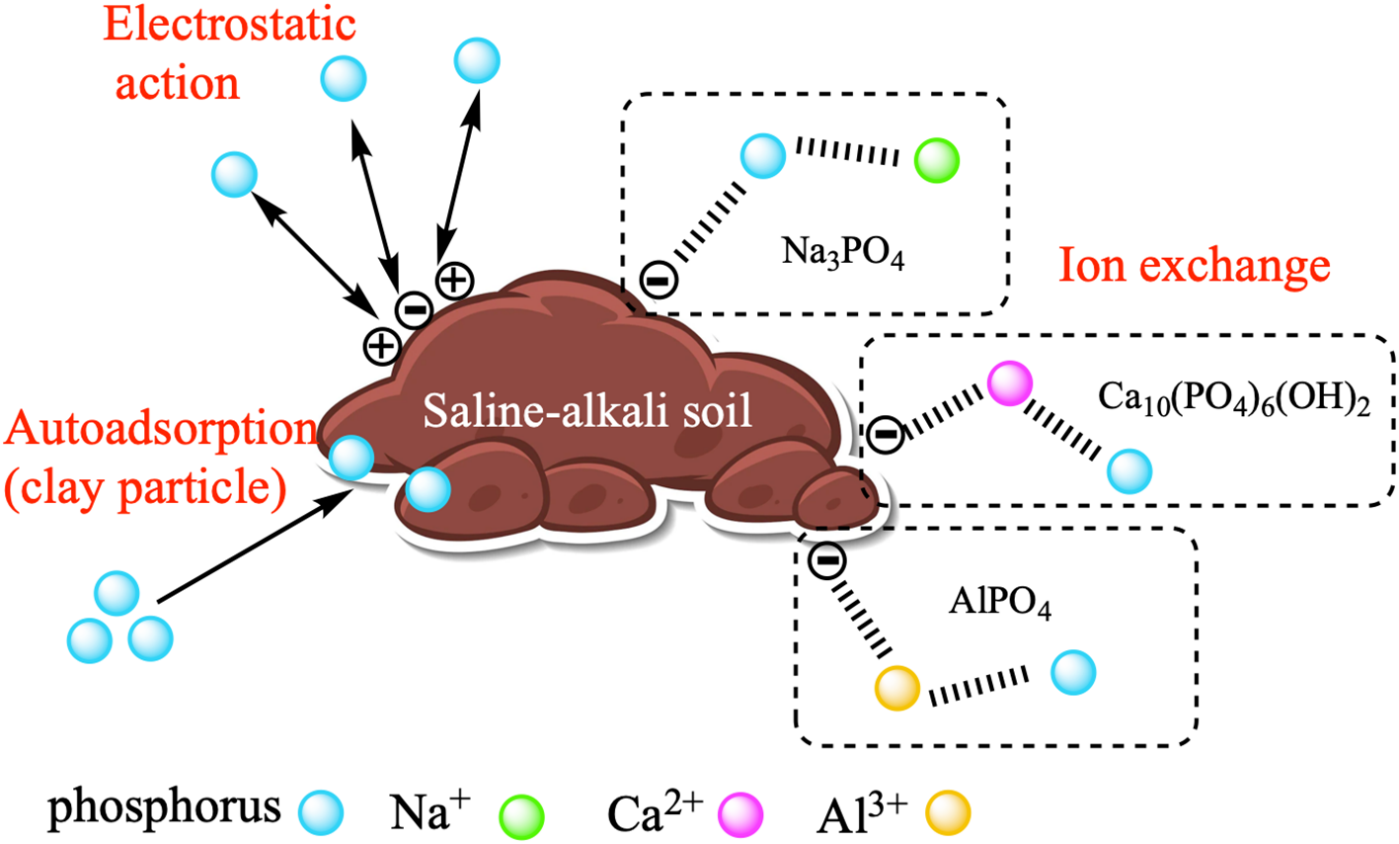
Mechanism of phosphorus adsorption in saline soils.

**Figure 6** summarizes the process of phosphorus adsorption by saline soils. The adsorption of phosphorus by saline-alkaline soil involves both physical and chemical adsorption. The adsorption performance is influenced by the physicochemical characteristics of the soil itself, along with the presence of SO_4_^2-^ and CO_3_^2-^. Typically, phosphorus is rapidly adsorbed onto the outer surface of immobilized soil particles. Once the outer surface reaches saturation, phosphorus then enters the pores within the saline soil until adsorption equilibrium is achieved (Jellali et al., 2011). In saline soils, the presence of sodium (Na^+^), calcium (Ca^2+^), and aluminum (Al^3+^) can combine with phosphate (PO_4_^3-^), suggesting that the primary mechanism of phosphorus adsorption in saline soils is electrostatic adsorption. PO_4_^3-^ can increase the adsorption capacity through the substitution with SO_4_^2-^ and CO_3_^2-^. As the pH rises, the OH- concentration also increases, combining phosphorus with OH-. When these two substances have opposite charges, binding takes place, resulting in electrostatic adsorption on the surface of saline soil and an increase in the level of adsorption.

### 3.4 Risk of phosphorus release from different salinized soils

The estimation of phosphorus adsorption capacity in soil was introduced, through the concepts of PSI and DPS (Bache and Williams, 1971; Williams et al., 1978). PSI exhibits a strong correlation with the maximum adsorption capacity of phosphorus, indicating the buffering effect of the adsorbent. A smaller PSI value indicates a weaker buffering effect, while a larger one suggests a stronger one (Xu et al., 2021). The smaller the value the lower the buffering capacity of the soil for phosphorus, and vice versa, the better the buffering effect (Xu et al., 2021). DPS can be used to evaluated the soil environmental capacity of phosphorus (Lauryssen et al., 2023), and to predict the potential of soil phosphorus loss, and the greater the DPS, the greater it means that most of the adsorption points on the soil surface that can adsorb phosphate in water have been occupied (Xu et al., 2022). According to Huang and colleagues, ERI of phosphorus was defined by (2004) as the ratio of DPS and PSI. Four levels of risk were assigned to potential phosphorus release: lower risk (ERI< 10%), moderate risk (10%< ERI< 20%), higher risk (20%< ERI< 25%), and high risk (ERI > 25%).

The ERI of the three adsorbents ranged from 5.40% to 17.72%, as indicated in **Table 8**. The ERIs of phosphorus followed the order: HS< MS< LS. As soil salinization progressed, the soil clay particle content increased, leading to an increase in exchange capacity. Consequently, the ERI of phosphorus adsorption strengthened with higher degrees of soil salinization. As the level of soil salinization rises, the soil’s clay content increases, leading to an increase in exchange capacity and a stronger ability to adsorb phosphorus. Consequently, as the soil salinization level increases, the ERI decreases. With the increase of temperature, ERI gradually decreased. The risk of phosphorus release and migration was more pronounced in the three salinized soils at lower temperatures.

**Table 8:** The ERI index of phosphorus in saline soils.

ERI	288K	298K	308K
LS	17.72%	14.96%	11.85%
MS	11.04%	8.19%	6.23%
HS	10.73%	6.18%	5.40%

**Figure 7:**
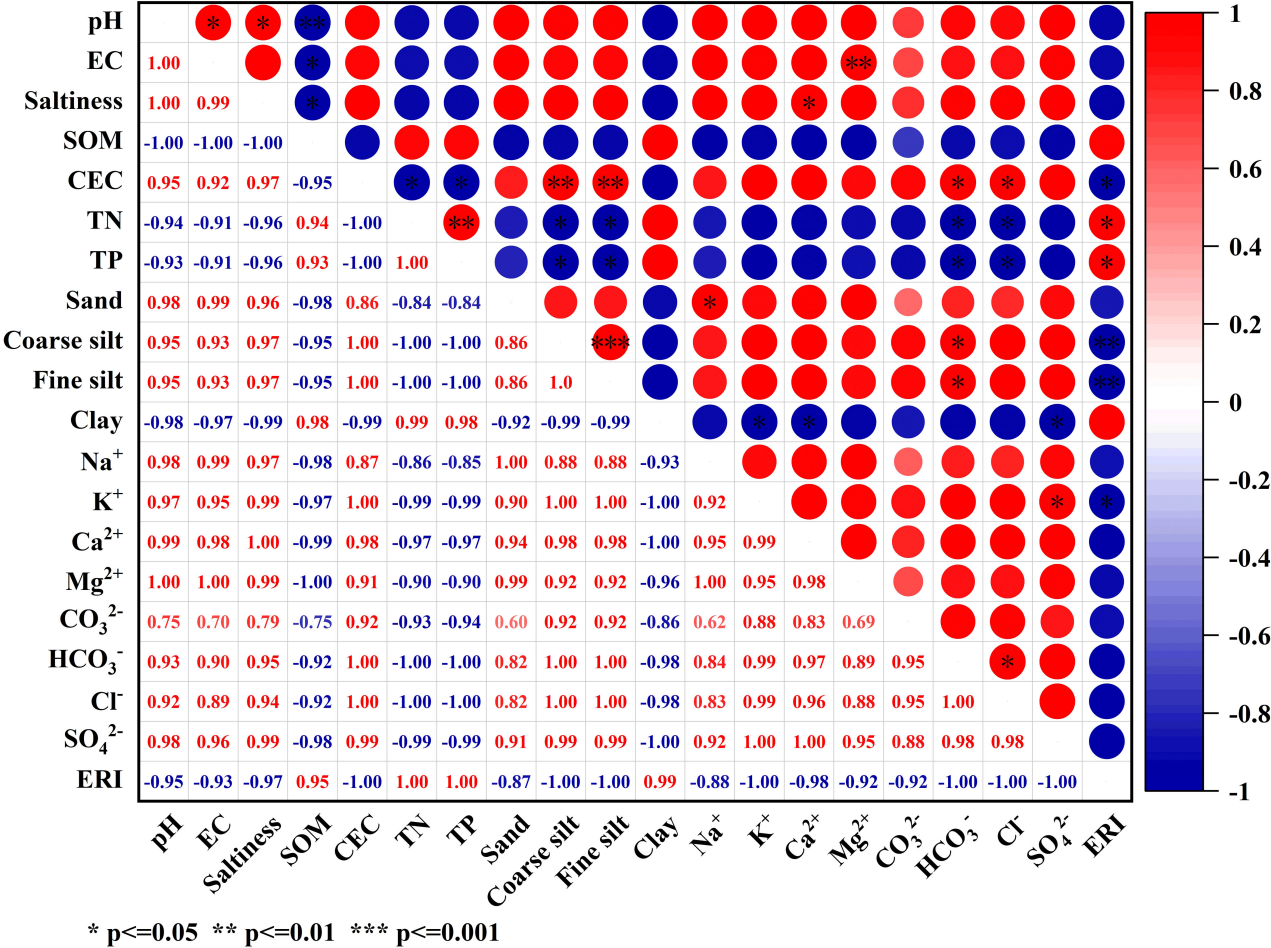
Correlation analysis of soil physicochemical properties and salt-based ions on phosphorus ERI.

**Figure 7** displays the correlation analysis between soil physicochemical properties and phosphorus ERI. ERI exhibited a highly significant negative correlation with Coarse silt and Fine silt (p ≤ 0.01), while TN and TP displayed a significant positive correlation with ERI (p ≤ 0.05). Additionally, K^+^ and CEC demonstrated a significant negative correlation with ERI (p ≤ 0.05). It has been shown that soil adsorption of phosphorus is mainly influenced by soil clay particle type and content, organic matter content, Ca, Mg content, Fe and Al oxides, and pH (Antoniadis et al., 2016). In amending saline soils, the application of aluminum sulfate increases P fixation by the soil. Out of the three soils affected by salinization in this research, the soil with severe salinization exhibited the highest concentration of SO_4_^2-^ and demonstrated the lowest likelihood of releasing P while immobilizing it. Due to the high levels of potassium (K^+^) and CEC in the saline soil, the potassium ions (K^+^) in the plasma would bind with phosphate ions (PO_4_^3-^), leading to an augmentation in phosphorus adsorption and a reduction in both transport activity and the likelihood of release. After analyzing PSI, DPS, and other parameters associated with phosphorus adsorption capacity in the three salinization soils, it was concluded that the LS exhibits the highest index of release risk. Therefore, it is crucial to prioritize monitoring the environmental transport risk in all three salinization soils, particularly during colder seasons.

## 4 Conclusion

In this paper, the adsorption mechanism of phosphorus was investigated by studying the adsorption characteristics and the risk of migration and release of phosphorus in different salinized soils. The experiment results showed that the three soils, which were affected by different levels of salinization, reached adsorption equilibrium after 720 min. The adsorption capacity of phosphorus varied among the soils, with LS having the lowest capacity, followed by MS, and HS having the highest capacity. Soil’s adsorption of phosphorus aligns better with the Pseudo-second-order model and Langmuir equation, involving spontaneous heat absorption and an increase in entropy. With pH increased the adsorption of phosphorus in the three salinized soils. According to the ERI, the order of salinized soil severity is as follows: LS > MS > HS. The risk of phosphorus release decreased as the temperature increased. ERI showed a significant correlation with TN, TP, CEC, K^+^, coarse silt, and fine silt. After analyzing PSI, DPS, and other parameters associated with phosphorus adsorption capacity in the three salinization soils, it was concluded that the LS exhibits the highest index of release risk.The results of the study can help to understand the risk of phosphorus transport and release in salinized soil and provide a theoretical basis for subsequent pollution prevention and control accordingly.

## Data availability statement

The raw data supporting the conclusions of this article will be made available by the authors, without undue reservation.

## Author contributions

YJ: Conceptualization, Formal analysis, Investigation, Writing – original draft. QY: Data curation, Methodology, Writing – original draft. TL: Investigation, Writing – original draft. YX: Supervision, Writing – original draft. XH: Resources, Visualization, Writing – review & editing. XM: Funding acquisition, Supervision, Writing – review & editing. YW: Visualization, Writing – original draft.
