# Supplementary material for: Phosphorus adsorption characteristics and release risk in saline soils: a case study of Songnen Plain, China
Source: Front Plant Sci. 2023 Dec 6;14:1302763. doi: 10.3389/fpls.2023.1302763 (PMC10731378; doi:10.3389/fpls.2023.1302763)
Supplement: Supplementary file 1 [file DataSheet_1.pdf]

Phosphorus adsorption characteristics and release risk in saline soils: a case study of Songnen Plain, China

Yan Jiang[1,†], Qiuliang Yan[2,†], Tonglinxi Liu[1], Yifan Xu[1,3], Xing Han[1,*], Xiulan Ma[1,*], Yujun Wang[1]

[1] College of Resources and Environment, Jilin Agricultural University, Changchun, China

[2] Institute of Animal Nutrition and Feed Sciences, Jilin Academy of Agricultural Sciences, Gongzhuling, China

[3] Jilin Huan Zhi Technology Co., LTD., Changchun, China

[†] These authors contributed equally to this work and share first authorship.

*** Corresponding author** : Xing Han, xingh@jlau.edu.cn; Xiulan Ma, lanma@jlau.edu.cn

Contents

P1: Contents

P2:Abbreviated list

P3：Method for fractional determination of phosphorus:

P4：Methods for determining physical and chemical properties of soil

P5~6:Table S1 Supplementary Equation

P7:Table S2: Comparison of phosphorus adsorption characteristics of different adsorbents

P8:References

Abbreviated list

Lightly saline soil : LS
Moderately saline soil : MS
Heavily saline soil : HS

Electric conductivity : EC
Soil organic matter : SOM
Cation exchange capacity : CEC
Total N : TN
Total P : TP

Phosphate sorption index : PSI
Degree of P saturation : DPS
Eutrophication risk index : ERI

Method for fractional determination of phosphorus:

The study used the Hedley classification method to classified the adsorbate for phosphorus, and determined Ca_2-P, Al-P, Org-P, Fe-P, O-Al-P, O- Fe-P, Ca_{10}-P.

(1) Ca_2-P: put 0.5 g of sample through 100 mesh sieve into a 50 mL centrifuge tube, add 25 mL of 0.25 mol/L NaHCO3 (pH 7.5), shake for 1 h, centrifuge, and wash the residue with 25 mL of ethanol;
(2) Al-P: Add 25 mL of 0.5 mol/L NH_4F (pH 8.5) to the residue in (1), shake for 1 hour, centrifuge, and wash the residue with 25 mL of saturated NaCl;
(3) Org-P: (2) Add 0.7 mol/L NaClO (pH8.05) to the residue in 25 mL boiling water bath for 30 minutes, cool and centrifuge, and wash the residue with 25 mL saturated NaCl;
(4) Fe-P: Take residue from (3) and add 25 mL of 0.1 mol/L NaOH-0.1 mol/L Na_2CO_3, shake for 2 h, then let stand for 16 h, shake for 2 h, centrifuge, and use 25 mL of saturated NaCl for the residue Wash 2 times;
(5) O-Al-P: Take residue from (4) and add 25 mL of 1 mol/L NaOH to a water bath at 85 °C for 1 h. After cooling, centrifuge, and wash the residue twice with 25 mL of saturated NaCl;
(6) O-Fe-P: Take residue from (5) and add 20 mL of 0.3 mol/L sodium citrate, stir evenly, add 0.5 g $Na_2S_2O_4$, 80 °C water bath, add 1 mol/L NaOH 5 mL, centrifuge, then wash residue twice with 25 mL saturated NaCl and retained.
(7) Ca10-P: Add 25 mL of 0.25 mol/L H_2SO_4 to the residue in (6), shake for 1 hour, and centrifuge.

The above experiments were set up with 3 groups of replications.

The fundamental physicochemical characteristics of the examined soil were assessed using the approach outlined in the publication 'Routine Analytical Methods of Soil Agrochemistry' (Bao et al., 1988), and the findings were presented in Table 1 and Table 2.

The above experiments were set up with 3 groups of replications.

Table S1 Supplementary Equation

	Equation
Phosphorus adsorption expression	$q_t = (C_0 - C_t)V/m$ (Eq.1)
Kinetic equation for phosphorus adsorption	Pseudo-first-order model ：$q_t = Q_{e,1}(1 - e^{-k_1 t})$ (Eq.2)
	Pseudo-second-order model：$q_t = \frac{Q_{e,2}^2 k_2 t}{1+Q_{e,2}k_2 t}$ (Eq.3)
Isothermal equation for phosphorus adsorption	Langmuir equation：$q_e = \frac{K_L q_m C_t}{1+K_L C_t}$ (Eq.4)
	Freundlich equation：$q_e = K_F C_t^{1/n}$ (Eq.5)
Thermodynamic parametric equations:	$K_F = \frac{(C_0 - C_e)V}{C_e m}$ (Eq.6)
	$\ln K_F = \frac{\Delta S}{R} - \frac{\Delta H}{RT}$ (Eq.7)
	$\Delta G = \Delta H - T\Delta S$ (Eq.8)

Phosphorus adsorption expression

Where: q_t is the amount of phosphorus adsorbed at time t (mg/kg); C_0 and C_t are the mass concentration of phosphorus in the adsorption system at the initial and t moments, respectively (mg/L); V is the volume (mL); m is the weight of the sample (g).

Kinetic equation for phosphorus adsorption

Where: q_t is the amount of phosphorus adsorbed at equilibrium (mg/g); q_e is the amount adsorbed at time t (mg/g); k_1 is the rate constant for The pseudo-first-order model (h); k_2 is the rate constant for pseudo-second-order model adsorption [g/(mg-h)].

The Pseudo-first-order model can be used as an explanation of the initial phase

of adsorption, while the Pseudo-second-order model is applicable to the entire adsorption phase of this experiment.

Isothermal equation for phosphorus adsorption

Where: q_e is the mass concentration of phosphorus in the solution system at equilibrium (mol/L); K_f is the Freundlich equilibrium constant; n is a constant characterizing the strength of adsorption; q_m is the theoretical maximum adsorption amount (mg/kg); and K_L is the adsorption affinity coefficient (L/mol).

Thermodynamic parametric equations:

Where: ΔG is the Gibbs free energy change (kJ/mol); K is the thermodynamic equilibrium constant, which can be fitted by the Langmuir equation (L/mol); R is the ideal gas constant, 8.314 [J/(mol-K)]; T is the reaction temperature (K); ΔH is the standard enthalpy change (kJ/mol); ΔS is the standard entropy change [kJ/(mol-K)]; the relationship between ΔG ～T, the slope and intercept of the line correspond to the values of ΔS and ΔH, respectively.

Table S2: Comparison of phosphorus adsorption characteristics of different adsorbents

Adsorbents	pH	SOM (g/kg)	Equilibrium concentration (mg/L)	Time (min)	q_m (mg/kg)	Temperature (°C)	References
Black calcium soil	8.23	8.60	0~150	0~1440	2057	298K	(Zhao et al., 2022)
Alfisol	6.51	17.28	20~300	0~2880	1248	-	(Jing et al., 2024)
Black soil	6.93	27.25	30~100	0~1440	1432	298K	(Xue et al., 2023)
surface soil layer from the farmers'fields India	7.53	0.59	0~80	0~2880	80	-	(Sharma et al., 2023)
Surface farm soil	6.74	23.90	5~45	0~1440	207	298K	(Amarh et al., 2021)
Red soil	6.30	-	0~150	482	570	296K	(Ilori et al., 2023)

References

Amarh, F., Voegborlo, R. B., Essuman, E. K., Agorku, E. S., Tettey, C. O., & Kortei, N. K. (2021). Effects of soil depth and characteristics on phosphorus adsorption isotherms of different land utilization types: Phosphorus adsorption isotherms of soil. Soil and Tillage Research, 213, 105139. https://doi.org/https://doi.org/10.1016/j.still.2021.105139

Bao S., Qin H., & Luo J. (1988). Soil agrochemical analysis. China Agricultural Press.

Ilori, A. O. A., Ogbonnaya, O. U., Asaolu, J. I., Shittu, O. S., & Fasina, A. S. (2023). Characterization of biochar and phosphorus adsorption in charnockite-originated soils. Journal of the Saudi Society of Agricultural Sciences, 22(1), 54-61. https://doi.org/https://doi.org/10.1016/j.jssas.2022.06.004

Jing, K., Min, X., Song, W., Xu, D., & Li, X. (2024). Effect of filling materials on reconstructed soil phosphorus adsorption and desorption in mining area. Soil and Tillage Research, 235, 105895. https://doi.org/https://doi.org/10.1016/j.still.2023.105895

Sharma, S., Sekhon, B. S., Singh, P., Siddiqui, M. H., & Kesawat, M. S. (2023). Response of biochar derives from farm waste on phosphorus sorption and desorption in texturally different soils. Heliyon, 9(9), e19356. https://doi.org/https://doi.org/10.1016/j.heliyon.2023.e19356

Xue, P., Hou, R., Fu, Q., Li, T., Wang, J., Zhou, W., Shen, W., Su, Z., & Wang, Y. (2023). Potentially migrating and residual components of biochar: Effects on phosphorus adsorption performance and storage capacity of black soil. Chemosphere, 336, 139250. https://doi.org/https://doi.org/10.1016/j.chemosphere.2023.139250

Zhao, D., Qiu, S.-k., Li, M.-m., Luo, Y., Zhang, L.-s., Feng, M.-h., Yuan, M.-y., Zhang, K.-q., & Wang, F. (2022). Modified biochar improves the storage capacity and adsorption affinity of organic phosphorus in soil. Environmental Research, 205, 112455. https://doi.org/https://doi.org/10.1016/j.envres.2021.112455
